# A 15-month evaluation of bond failures of orthodontic brackets bonded with direct versus indirect bonding technique: a clinical trial

**DOI:** 10.1186/s40510-014-0070-9

**Published:** 2014-12-30

**Authors:** Anna Menini, Mauro Cozzani, Maria Francesca Sfondrini, Andrea Scribante, Paolo Cozzani, Paola Gandini

**Affiliations:** Unit of Orthodontics and Paediatric Dentistry, Department of Clinical, Diagnostic and Paediatric Sciences, School of Dentistry, University of Pavia, Pavia, Italy; Department of Surgical Science, School of Dental Medicine, University of Cagliari, Cagliari, Italy; Private Practice, La Spezia, Italy

**Keywords:** Indirect bonding technique, Bond failure, Clinical trial

## Abstract

**Background:**

The purpose of this clinical longitudinal study was to investigate the effectiveness of indirect bonding technique evaluating the number of bond failures which occurred during treatment.

**Methods:**

Fifty-two patients were selected and divided into two groups: group A (33 patients) bonded with the direct technique and group B (19 patients) bonded with the indirect technique. The number and date of bracket failure were recorded for over 15 months. Moreover, also the effect of crowding level on bracket failures was calculated. Statistical analysis was performed by means of *t*-test, Kaplan-Meier survival estimates and chi-squared test.

**Results:**

No statistically significant differences were found in the total bond failure rate between direct and indirect techniques, also when comparing the upper and lower arches. The only significant difference was found comparing the posterior segment of the lower arches, in which a higher percentage of detachments were recorded in group B, bonded with the indirect technique. Moreover, no significant differences between direct and indirect bonding were found when evaluating crowding level.

**Conclusions:**

Orthodontic practitioners can safely use the indirect bonding technique, even in patients with severe crowding, because it does not influence the adhesive quality and the bracket survival rate.

## Background

In orthodontics, indirect bonding technique offers numerous advantages over direct bonding [1], such as chair time saving, a more precise bracket placement [2–4] and removal of flash to the bracket bases [5,6] which can promote plaque and calculus formation [7].

Indirect bonding technique involves a two-stage procedure. The first stage is carried out in a laboratory, where brackets are positioned and attached to a plaster model of the patient's teeth. The second stage is clinical: when the brackets are transferred from the cast model to the patient's mouth with a tray and bonded to the etched enamel.

Indirect bonding has become increasingly popular since the method was first described in detail by Silverman and Cohen [8,9] in 1972. Different indirect bonding techniques have been proposed, with different preparations of the bracket base (standard or customized), transfer mask type (single jigs or full arch) and transfer tray material (acrylic resin, silicone, thermo-printed material) [10–12].

Many studies have been conducted in order to test the indirect technique effectiveness. In fact, only few reports evaluated the clinical reliability of the indirect bonding technique compared with the conventional bonding technique.

Accordingly, the purpose of the present study is to test the efficacy of indirect bonding compared with conventional direct bonding, evaluating the number of bond failures which occurred during treatment; the observation period was 15 months for both groups. The null hypothesis of the study was that there is no significant difference in bracket failure rates between the two bonding techniques, also when comparing the upper and lower arches and the anterior and posterior segments.

## Methods

A total of 52 patients treated with fixed orthodontic appliance were included in this study. All of them had to satisfy the following inclusion criteria:

- permanent dentition
- no dental morphogenesis anomalies
- no enamel anomalies
- no presence of vestibular reconstruction
- no genetic syndromes connected with oral cavity anomalies
- no need of adjunctive orthodontic device (facial mask, forsus, etc.)

No restriction was applied in respect of age, gender, type of malocclusion and grade of crowding.

For each patient, the following were recorded: the date of bonding, age at the start of treatment, gender, value of irregularity index [13] (calculated on the initial model cast) and type of malocclusion. A formal sample size calculation was not undertaken.

Fifty-two patients agreed to participate and were divided into two groups: 33 patients in group A (18 females and 15 males, mean age = 20.75 years) bonded with the direct technique and 19 patients in group B (9 females and 10 males, mean age = 25 years) bonded with the indirect technique. Informed consent was obtained from all the patients before starting the therapy, and the study was conducted in accordance with the Declaration of Helsinki. Data analysts were blinded; however, blinding of the operators was not possible.

Stainless steel brackets and molar tubes 0.018″ × 0.025″ and 0.022″ × 0.028″ (AO, 1714 Cambridge Avenue, Sheboygan, WI, USA) and the same adhesive system (Transbond XT, 3M, Monrovia, CA, USA) were used for both groups.

All patients had professional oral hygiene 3 days before bonding, and then both arches were bonded, including the first molars.

All patients were provided with oral instruction for maintenance of their fixed appliance. Checkup was carried out every 4 weeks. The patients were recommended to inform the dentist immediately if suspecting a detachment. Detachment date was registered, and the bracket was replaced with a new one; in group B, a section of the transfer tray used for the initial bonding has been used for repositioning when necessary.

A total of 1,248 brackets were positioned: 792 attachments were fixed with the direct technique while the remaining 456 with the indirect technique. The observation period was 15 months.

### Direct technique

All teeth were cleaned with water and fluoride-free pumice for at least 30 s and then dried with an oil-free air syringe. The enamel was then etched for 30 s with 37% orthophosphoric acid (etching gel, 3M, Monrovia, CA, USA), and the Primer (Transbond XT, 3M, Monrovia, CA, USA) was applied with a small brush and spread with oil-free compressed air. The composite (Transbond XT, 3M, Monrovia, CA, USA) was applied on the bracket base, and the attachment was positioned on the tooth surface. Composite excess was removed with a probe before polymerization. The composite was polymerized with a LED lamp (Opticore L3; Marslev Byvej, Denmark) for 80 s per bracket (20 s for side: mesial, distal, occlusal and gingival).

### Indirect technique

**Figure 1:**
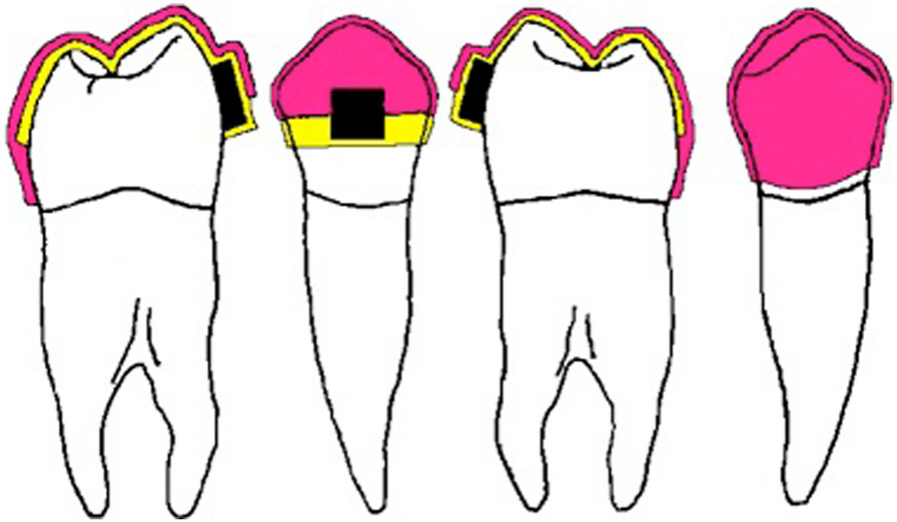
**Soft tray extending buccally to cover the entire bracket and rigid tray extending only to the bracket slot.** The soft tray (yellow) extends buccally to cover the entire bracket, holding it in place while covering half of the clinical crown on the lingual side. The rigid tray (pink) extends only to the bracket slot to retain the bracket without impeding tray removal during the clinical phase.

For indirect bonding, the modified Fantozzi technique [5,14] was used, which consists of two transparent trays: one for etching and one for bracket transfer. Each tray is divided into three sections: one anterior (incisors and canines) and two posterior (premolars and first molars). The transfer tray is made of two layers: the inner one is soft and thin and holds the bracket completely, while the extern is rigid and assures a precise position during the trays transfer from the cast to the oral cavity (Figure 1). The composite is positioned on the bracket base during the laboratory phase [14].

The clinical phase was carried out as follows: the etching mask was placed over the cleaned facial surface of the appropriate teeth. The etchant was applied for 30 s. The mask was removed, the teeth rinsed thoroughly for about 10 s and then dried. A layer of primer (Transbond XT) was applied both on the etched surface and the bracket base inside the transfer tray; the trays were fitted in the mouth and light-cured for 20 s each on the buccal, distal, mesial and occlusal sides, for a total of 80 s per bracket. The transfer trays were removed using a probe.

### Statistical analysis

The statistical analysis was performed using the Software Stata 9 (Stata, College Station, TX, USA).

Differences in bond failure frequency in groups A and B were analysed using a Fisher exact test. To evaluate the time of detachment, Kaplan-Meier survival curves were obtained using a log-rank test.

Chi-squared test was used to analyse the detachment in the upper jaw compared to the lower jaw, and when considered, each arch splits into anterior and posterior sections.

To analyse irregularity index, samples were divided into two groups (irregularity index <4 mm and irregularity index >4 mm) and chi-squared test was applied. Significance level was set at *P* < 0.05.

## Results

### Sample description

In group A, of 33 patients, 792 brackets were bonded using the direct technique. Nine patients presented a class I malocclusion, 18 a class II and 6 a class III. The mean value of irregularity index was 2.58 mm (DS 2.57 mm); 28 detachments were reported.

In group B, of 19 patients, 456 brackets were bonded using the indirect technique. Nine patients presented a class I malocclusion, 8 a class II and 2 a class III. The mean value of irregularity index was 3.58 mm (DS 2.52 mm); 26 detachments occurred.

### Bond failure

No significant differences were found between direct and indirect bonding (*P* < 0.05) (Table 1).

**Table 1:** **The number and percentage of detachments in groups A and B**

	**Bonded brackets**	**Bond failure**	**Percentage**	**Fisher'** **s exact test**
Group				
A	792	28	3.54	ns
B	456	26	5.70	
Total	1,248	54	4.32	

Group A: bonded with the direct technique; group B: bonded with the indirect technique. ns, not significant.

**Figure 2:**
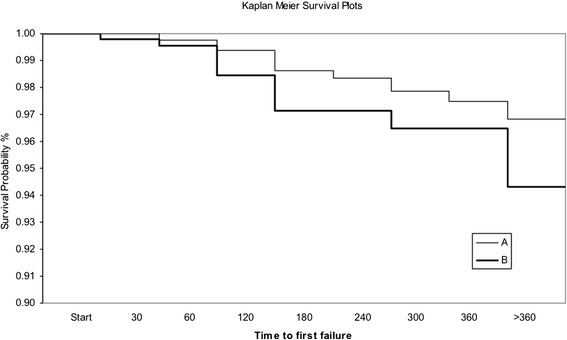
**Kaplan-Meier survival plots.** **A:** Direct Bonding, **B:** Indirect bonding.

Kaplan-Meier survival plots showed no significant differences during the observation period between group A and group B (Figure 2) (hazard ratio: 0.61; confidence interval 95%: 0.34 to 1.04; log-rank test: *P* = 0.0699).

Bond failure comparing the upper and lower arches was also investigated (Tables 2 and 3). No differences in the upper jaws were found between two techniques, while a significantly greater number of detachment occurred in the lower arch (9.64% of the total bonded bracket).

**Table 2:** **Comparison between groups A and B of detachment number in the upper arch**

	**Upper**		
	**Total upper arch**	**Upper anterior section**	**Upper posterior section**
Group			
A	396-11	198-2	198-9
	2.77%	1.01%	4.54%
B	228-4	114-1	114-3
	1.75%	0.88%	2.63%
Total	624-15	312-3	312-12
	2.40%	0.96%	3.85%
Chi-squared test	ns	ns	ns

Group A: bonded with the direct technique; group B: bonded with the indirect technique. Detachment number is considered in total and in sections. The number of bonded brackets and bond failure are reported and below the detachment percentage. ns, not significant.

**Table 3:** **Comparison between groups A and B of detachment number in the lower arch**

	**Lower**		
	**Total lower arch**	**Lower anterior section**	**Lower posterior section**
Group			
A	396-17	198-6	198-11
	4.29%	3.03%	5.56%
B	228-22	114-2	114-20
	9.64%	1.75%	17.54%
Total	624-39	312-8	312-31
	6.25%	2.56%	9.94%
Chi-squared test	*P* < 0.05	ns	*P* < 0.01

Group A: bonded with the direct technique; group B: bonded with the indirect technique. Detachment number is considered in total and in sections. The number of bonded brackets and bond failure are reported and below the detachment percentage. ns, not significant.

The number of detachments in the anterior (including incisors and canines) and posterior (including molars and bicuspids) sections was also investigated for both upper and lower arches (Tables 2 and 3). No significant differences were found in the upper jaws neither for the anterior nor for the posterior segments in group A as compared with group B. As regards the lower jaws, in the posterior section, chi-squared test showed that the percentage of detachments was greater in group B (17.54%), bonded with the indirect technique, than in group A (5.56%) (*P* < 0.01).

### Irregularity index

*t*-test revealed that the two groups were homogeneous as regards the irregularity index (I.I.) value.

It was also decided to subdivide the sample on the strength of the I.I. value, in order to investigate the differences of bond failure in patients with various crowding levels.

Patients with severe crowding level were considered if the I.I. values were ≥4 mm and light crowding level when the I.I. values were <4 mm. Chi-squared test showed no significant differences in the number of bond failure between the two groups (Table 4).

**Table 4:** **Comparison of detachment number in groups A and B with a high/low value of I.I.**

**Group**	**Number of bond**	**Number of failure**	**Percentage**	**Chi-squared test**
I.I. < 4 mm				
A	528	22	4.17	ns
B	192	13	6.77	
I.I. ≥ 4 mm				
A	264	6	2.27	ns
B	264	13	4.92	

Group A: bonded with the direct technique; group B: bonded with the indirect technique. ns, not significant.

## Discussion

As reported in the results, the null hypothesis of the study was partially accepted. In fact, the present investigation demonstrated that when using the indirect technique, the bond failure rate is not significantly different than when the direct technique is used.

These results are in agreement with Deahl et al. [15] and Thiyagarajah et al. [16] that found no difference in detachment rate in the two techniques. Nevertheless, we can notice that the percentage of bond failure reported in the present study (3.54% for direct technique, 5.79% for indirect one) is higher for both techniques than the percentage reported by Deahl (1.17% ± 3.62% and 1.21% ± 3.81%, respectively) as well as those reported by Thiyagarajah (2.9% for direct technique, 2.2% for indirect one). The reasons of this difference can be found in the numerous differences in the study drawing: type of bracket and adhesive system, procedure used for the transfer tray confection, number of patients included in the sample and study design. It is important to consider that in our investigation, the first molar tubes were included in all statistical analysis and these attachments presented a great number of bond failure: on 28 total detachments in the direct technique, 13 were molar tubes, while 8 were the molar tube detachments on 26 of the total bond failure observed for the indirect technique.

The detachment rate obtained in our study for patient bonded with the indirect technique is similar to the findings of Read and O'Brien [17] that investigated the failure rate in patients bonded with the indirect technique using a new visible light-cured adhesive (Opalux, I.C.I. Dental, Macclesfield, UK). On the other hand, Read did not found any differences in comparing anterior and posterior sections. As regards the direct technique, the results are similar to others reported by various authors: Mirabella et al. [18] found a bond failure rate of 2.95%, thus showing a significant difference between the upper and lower arches (1.67% and 4.35%, respectively); Varlik and Demirbaş [19] reported a percentage of 3.7%; and Pasquale et al. [20] of 4.1%.

Romano et al. [21] recorded a lower percentage value (1.57%), even if it is proper to consider that he did not include the first molars in his study, and the observation period was 6 months.

Shear bond strength of brackets bonded with the indirect technique has been tested *in vitro*, and comparison with direct bonding shows no significant difference between the two techniques [22–24].

A direct comparison among the studies present in the literature is difficult, as numerous differences and variances among various investigations (type of bracket and adhesive [25,26], observation period, sample size, etc.) are present.

Kaplan-Meier survival plots showed no statistically significant differences in failure rate between brackets bonded with direct or indirect technique. A longer period of examination could reveal if the discrepancy, actually not significant, between the two curves is going to increase over time, thus demonstrating a difference in survival rate of brackets bonded directly or indirectly, or if the results obtained in the present research are confirmed also after a longer observation time.

Moreover, in the present investigation, different results were found in the percentage of failure when comparing the upper and lower arches as well as the anterior and posterior segments.

In fact, as regards the upper jaw, no significant differences were found in groups A and B when considering full arch neither when considering sectors separately. Statistically significant results (*P* < 0.01) were observed in the posterior segment of the lower arch, with a higher failure rate in group B (17.54%) when compared to group A (5.56%).

Gender, age and type of malocclusion were not evaluated in the present work. Conflicting reports are present in the literature concerning these variables: some authors found significant differences in bond failure rate in patients with different malocclusions [27], age [28] and gender [29]. Others found the same results in male and female [27,30,31], patients with different age [30,31] and malocclusion [29,31].

To analyse the irregularity index, group A and group B were splitted in two subgroups: with high I.I. values (≥4 mm) or low I.I. values (<4 mm). No differences were found between direct and indirect techniques also when evaluating the irregularity index values. Crowding severity seems not to be a variance influencing risk of detachment. The increase of I.I. values does not correspond to an increased percentage of failure rates.

This suggests that the use of transfer trays offers a good adhesion quality also in the case of severe crowding, as much as the direct bonding technique.

Indirect technique does not seem to be less efficient than the direct one in terms of detachment percentage; neither bond failure seems to be influenced by crowding level. Therefore, the indirect technique can be used safely also in patients presenting crowding and rotations in the anterior segments or when malpositioned elements could seem an obstacle for a correct fitting of the trays.

## Conclusions

In the present clinical study, there were no statistically significant differences in the total bond failure rate between direct and indirect techniques. No statistically significant differences in the percentage of detachment were recorded between the two different bonding techniques when comparing the upper and lower arches.

The only difference in bond failure rate was observed comparing the posterior segments of the lower arch, in respect of which was found a significantly greater number of detachment in group B, bonded with the indirect technique. No difference in bond failure can be associated to crowding level.

Orthodontic practitioners can safely use the indirect bonding technique, even in patients with severe crowding, because it does not influence the adhesive quality and the bracket survival rate.

## References

[1] Kalange JT (2004). Indirect bonding: a comprehensive review of the advantages. World J Orthod.

[2] White LW (1999). A new and improved indirect bonding technique. J Clin Orthod.

[3] Fortini A, Giuntoli F, Franchi L (2007). A simplified indirect bonding technique. J Clin Orthod.

[4] Hoge TM, Dhopatkar AA, Rock WP, Spary DJ (2001). The Burton approach to indirect bonding. J. Orthod..

[5] Cozzani M, Menini A, Bertelli A. **Etching masks for precise indirect bonding.** *J Clin Orthod.* **44:**326–330. 20831102

[6] Kalange JT, Thomas RG (2007). Indirect bonding: a comprehensive review of the literature. Semin. Orthod..

[7] Sinha PK, Nanda RS, Ghosh J (1995). A thermal-cured, fluoride-releasing indirect bonding system. J Clin Orthod.

[8] Silverman E, Cohen M (1972). A universal direct bonding system for both metal and plastic brackets. Am. J. Orthod..

[9] Silverman E, Cohen M (1975). A report on a major improvement in the indirect bonding technique. J Clin Orthod.

[10] Dalessandri D, Dalessandri M, Bonetti S, Visconti L, Paganelli C (2012). Effectiveness of an indirect bonding technique in reducing plaque accumulation around braces. Angle Orthod..

[11] Zachrisson B, Brobakkeen B (1978). Clinical comparison of direct versus indirect bonding, with different bracket types adhesives. Am. J. Orthod..

[12] Aguirre MJ, King GJ, Waldrom MJ (1982). Assessment of bracket placement and bond strength when comparing direct bonding technique. Am. J. Orthod..

[13] Little RM (1975). The irregularity index: a quantitative score of mandibular anterior alignment. Am. J. Orthod..

[14] Fantozzi F. **Brackettaggio indiretto: fasi di laboratorio sulla costruzione personalizzata di trasbrackets e mascherine per la mordenzatura.** *Boll Inform Ortod.* 1997; **56:**38–45.

[15] Deahl ST, Salome N, Hatch JP, Rugh JD (2007). Practice-based comparison of direct and indirect bonding. Am J Orthod Dentofacial Orthop.

[16] Thiyagarajah S, Spary DJ, Rock WP (2006). A clinical comparison of bracket bond failures in association with direct and indirect bonding. J. Orthod..

[17] Read MJ, O’Brien KD (1990). A clinical trial of an indirect bonding technique with a visible light-cured adhesive. Am J Orthod Dentofacial Orthop.

[18] Mirabella D, Spena R, Scognamiglio G, Luca L, Gracco A, Siciliani G (2008). LED vs halogen light-curing of adhesive-precoated brackets. Angle Orthod..

[19] Varlik SK, Demirbaş E (2009). Effect of light-cured filled sealant on the bond failure rate of orthodontic brackets in vivo. Am J Orthod Dentofacial Orthop.

[20] Pasquale A, Weinstein M, Borislow AJ, Braitman LE (2007). In-vivo prospective comparison of bond failure rates of 2 self-etching primer/adhesive systems. Am J Orthod Dentofacial Orthop.

[21] Romano FL, Valério RA, Gomes-Silva JM, Ferreira JT, Faria G, Borsatto MC (2012). Clinical evaluation of the failure rate of metallic brackets bonded with orthodontic composites. Braz Dental J.

[22] Yi GK, Dunn WJ, Taloumnis LJ (2003). Shear bond strength comparison between direct and indirect bonded orthodontic brackets. Am J of Orthod and Dent Orthop.

[23] Linn BJ, Berzins DW, Dhuru VB, Bradley TG (2006). A comparison of bond strength between direct- and indirect-bonding methods. Angle Orthod..

[24] Swetha M, Pai VS, Sanjay N, Nandini S (2011). Indirect versus direct bonding - a shear bond strength comparison: an in vitro study. J. Contemp. Dent. Pract..

[25] Montasser MA, Taha M (2014). Effect of enamel protective agents on shear bond strength of orthodontic brackets. Prog. Orthod..

[26] Elsaka SE, Hammad SM, Ibrahim NF (2014). Evaluation of stresses developed in different bracket-cement-enamel systems usig finite element analysis with in vitro bond strength tests. Prog. Orthod..

[27] Millett DT, McCluskey LA, McAuley F, Creanor SL, Newell J, Love J (2000). A comparative clinical trial of a compomer and a resin adhesive for orthodontic bonding. Angle Orthod..

[28] Millett DT, Gordon PH (1994). A 5-year clinical review of bond failure with a no-mix adhesive (Right on®). Europ J Orthod.

[29] Shammaa I, Ngan P, Kim H, Kao E, Gladwin M, Gunel E, Brown C (1999). Comparison of bracket debonding force between two conventional resin adhesive and a resin-reinforces glass ionomer cement: an *in vitro* an *in vivo* study. Angle Orthod..

[30] Marcusson A, Norevall LI, Persson M (1997). White spot reduction when using glass ionomer cement for bonding in orthodontics: a longitudinal and comparative study. Europ J Orthod.

[31] Millett DT, Hallgren A, Cattanach D, McFadzean R, Pattison J, Robertson M, Love J (1998). A 5-years clinical rewiew of bond failure with a light-cured resin adhesive. Angle Orthod..

